# Remote Sensing Image Ship Matching Utilising Line Features for Resource-Limited Satellites

**DOI:** 10.3390/s23239479

**Published:** 2023-11-28

**Authors:** Leyang Li, Guixing Cao, Jun Liu, Xiaohao Cai

**Affiliations:** 1 School of Computer Science and Engineering, Northeastern University, 169 Chuangxin Road, Shenyang 110169, China; castcao13579@163.com (G.C.); liujun@cse.neu.edu.cn (J.L.); 2 School of Electronic and Computer Science, University of Southampton, Highfield Street, Southampton SO17 1BJ, UK; x.cai@soton.ac.uk

**Keywords:** remote sensing, image matching, line feature, ship, satellite

## Abstract

The existing image matching methods for remote sensing scenes are usually based on local features. The most common local features like SIFT can be used to extract point features. However, this kind of methods may extract too many keypoints on the background, resulting in low attention to the main object in a single image, increasing resource consumption and limiting their performance. To address this issue, we propose a method that could be implemented well on resource-limited satellites for remote sensing images ship matching by leveraging line features. A keypoint extraction strategy called line feature based keypoint detection (LFKD) is designed using line features to choose and filter keypoints. It can strengthen the features at corners and edges of objects and also can significantly reduce the number of keypoints that cause false matches. We also present an end-to-end matching process dependent on a new crop patching function, which helps to reduce complexity. The matching accuracy achieved by the proposed method reaches 0.972 with only 313 M memory and 138 ms testing time. Compared to the state-of-the-art methods in remote sensing scenes in extensive experiments, our keypoint extraction method can be combined with all existing CNN models that can obtain descriptors, and also improve the matching accuracy. The results show that our method can achieve ∼50% test speed boost and ∼30% memory saving in our created dataset and public datasets.

## 1. Introduction

**Figure 1:**
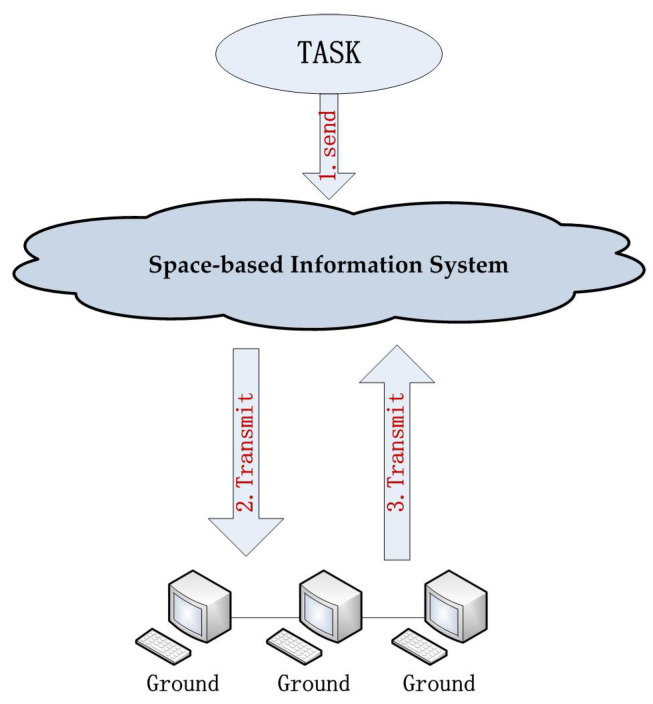
A space-based information system.

In recent years, space-based information systems have become an important part of technology for image processing and computer vision [1,2]. They can acquire diverse multimodal modern remote sensing data and process them intelligently. At present, the workflow of most of the space-based information is time-consuming, especially under a complex task. The satellite needs to transmit the data to the computer on the ground when it receives a mission, and then the result is sent back to the satellite after completing the task on the ground, see Figure 1. This complicated process is attributed to the inability of running large algorithms on the satellite itself because of its limitations in computation power in that the satellite needs to control its weight, power and heat dissipation within a low value such that it can run stably in space. Therefore, the main focus of this work is to develop an efficient and effective remote sensing image matching approach for the particular use on the satellite.

Ship is a very important object in remote sensing computer vision tasks. It can be used in different applications, including e.g., monitoring [3,4] and real-time quantity statistics [5]. At present, most algorithms use local features to match two images after the introduction of SIFT [6]. Methods based on neural networks e.g., SuperPoint [7] and based on gradient e.g., SURF [8] are common solutions for extracting local features. In the remote sensing image matching task, a robust local feature has been proven to be helpful, and different strategies have been proposed to attain it. For example, the work in [9] proposed a novel pixelwise feature representation using orientated gradients of images, which achieved superior performance in image matching and is computationally efficient; the work in [10] used the phase congruency instead of image intensity for local feature detection, and considered the number and repeatability of local features.

**Figure 2:**
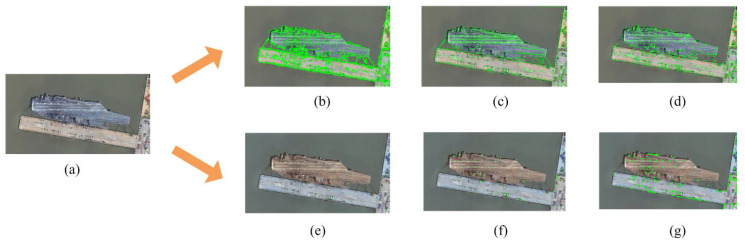
Local features extracted by different methods. (**a**): original image; (**b**,**e**–**g**): point features extracted by SIFT, SUFR, ORB and Harris, respectively; (**c**): line features extracted by LSD; and (**d**): point features extracted by the proposed approach using the endpoints of line segments.

Except for point features used in image matching tasks, line feature has extra advantages over the point features. For example, the line feature can represent structural information more effectively and is more common in ship and many other objects. Moreover, it is more robust to environmental changes, see Figure 2 which depicts the keypoints obtained using the line features and the comparison to other point detectors (e.g., SIFT, SUFR, ORB and Harris). In this sense, although the line feature might be more difficult to parameterize than point features, it can carry more environmental information in diverse settings. Recent research in computer vision using line features has attracted lots of attention, and some works used line features for the application in remote sensing images (see e.g., Section 2.1 and Section 3.1). They share a common limitation, i.e., the power cost and parameters used are not considered seriously, and therefore they are hard if not impossible to be used on resource-limited satellites. To address this challenge, we aim to build a method that can use few but precise keypoints to realise powerful matching results.

In this paper, we propose a new remote sensing image ship matching approach by utilising the developed novel keypoint extraction strategy named line features based keypoint detection (LFKD) for particular use on the satellite. The proposed method addresses the wrong matching caused by dense keypoints and improves the efficiency of the algorithm. In addition, our method takes the initiative in focusing on object matching rather than the whole image matching, which can give more attention to important objects within images. Our main contributions are as follows:

(1) We propose a keypoint extraction method, utilising line features to assist the keypoint selection. The keypoints selected in this way are sparse and more reasonable/precise, which aid to improve the accuracy and efficiency of the algorithm.
(2) We use a function to crop images during the matching process, which achieves end-to-end matching.
(3) We create new remote sensing image dataset about three kinds of ships (i.e., aircraft carrier, cargo ship and submarine), with variations in light, angle and size. Using this created remote sensing data, we experimentally show that too many dense keypoints are generally unnecessary for this image matching task partly because the fundamental matrix for image matching can be calculated with only eight points [11].

We demonstrate that our way can enhance the matching accuracy and boost the computation efficiency. Experiments also show that our method is more effective and low-consuming, ensuring it meets the conditions for running on satellites.

The remainder of this article is organized as follows. Section 2 reviews the related work. Section 3 details the proposed method. The experimental results and detailed comparisons with discussion are given in Section 4. Finally, we conclude in Section 5.

## 2. Related Work

### 2.1. Overview of Feature Detectors

Feature detectors are algorithms or methods used in computer vision and image processing to identify and extract specific features or patterns from images or visual data. Detected local features represent specific semantic structures in an image and can be divided into point feature [12,13,14,15] and line feature [16,17,18]. Due to the strong value of local features, some feature detectors have been designed. For instance, blob detectors Laplacian of Gaussian (LoG) [19] and the Difference of Gaussians (DoG) [20] identify regions of an image with uniform intensity or color (typically representing objects or regions of interest); corner detectors like Harris detector [21] and Shi-Tomasi detector [22] identify locations in an image where two or more edges intersect or change direction, with applications in image registration, tracking and object detection; edge detectors [23] identify sharp changes in pixel intensity (representing edges or boundaries between different regions in an image); and scale-invariant feature detectors identify features that are invariant to changes in scale, rotation, and illumination.

Parallel to the above traditional handcrafted methods, machine learning and deep learning-based methods have gained remarkable attention in recent years. For interest point detection, decision trees [24] have also been applied successfully to identify the interest points and the corners. The work in [14] extended the randomized trees [25] with LoG filters to detect the points at multi-scale levels. Later, machine learning methods have been combined with some generic algorithms to extract features. The research work has been carried out in literature based on hybrid methods. Strecha et al. [26] trained classifiers with WaldBoost learning to select feature points more relevant to the specific task. Hartmann et al. [27] trained classifiers to predict the most matched descriptors. By adding a prediction to the pipeline structure, the matching score has been improved without losing features. Verdie et al. [28] proposed a temporally invariant learned detector to detect repetitive keypoints. The accuracy of machine learning methods highly depends on the data representation. If the data is not represented correctly, the accuracy decreases.

According to the recent research, the most popular local feature in image matching task is point feature. The keypoints are easy to extract and define with a simplified form compared with the line features, and thus the work related to point feature is the most popular research area in a decade.

### 2.2. Image Matching Models

**Figure 3:**
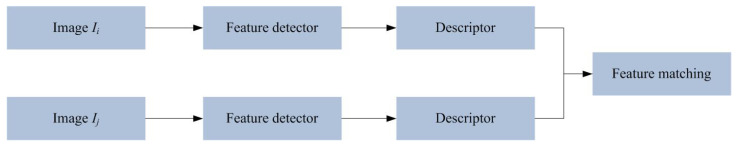
Workflow of the image matching task.

Image matching can be divided into three stages: feature detection, feature description, and feature matching. The process of this task is as follows. Firstly, the feature extractor extracts the features of interest from the image, which will be used for matching. Secondly, feature description refers to transforming each region around the detected keypoints into a more compact and stable descriptor. Finally, feature matching is conducted by efficiently searching for possible matching candidates from other images and establishing a match between two images. The workflow of image matching can be depicted as Figure 3.

The matching task aims to establish the correct image correspondences between two images with or without using the feature detection and/or description. Over the past decades in the image matching area, existing methods can be roughly classified into two categories, i.e., area-based and feature-based methods. Area-based methods aim for image registration and establish dense pixel correspondences by directly using the pixel intensity of the entire image. For example, the correlation-like methods [29,30], which are regarded as a classical representative in area-based methods, correspond two images by maximising the similarities of two sliding windows for the image matching task; and the domain transformed methods based on Fourier shift theorem [31,32] and the Walsh transform-based methods [33,34] tend to obtain matches on the basis of converting the original image domain into another. Feature-based methods use keypoints and their local descriptors extracted from the image pairs to match two images. The type of methods based on feature detectors and descriptors becomes a mainstream principle. For example, the work in [35] developed a robust and accurate multi-source matching with deformed contour segment similarity (DCSS); a CNN-based feature detector aiming to obtain a strong descriptor was proposed in [36]; and the works in [37,38] are about real-time image matching with improved accuracy and speed achieved. On the whole, the feature-based methods can extract distinctive features from images and then match these features across different images.

### 2.3. Image Matching in Remote Sensing

The image matching task in remote sensing has made progress based on the above-mentioned image matching pipeline. The technical framework [39] with affine invariant feature extraction and RANSAC was proposed for achieving higher correctness. The work in [40] designed a feature learning way based on two-branch networks to transform the image matching into a two-class classification problem. The method in [41] shows a novel descriptor for illumination-robust remote sensing image matching. Some detection algorithms for remote sensing images were modified based on object matching to address the challenges raised by the registration accuracy, the radiometric correction accuracy, and the classification threshold for difference images [42,43]. The work in [44] proposed a cross-modal feature description matching network using a self-attention module and cross-fusion module to consider the similarity of cross-modal features for obtaining better descriptors. The work in [45] designed a visualized local structure generation-Siamese attention network, which is an effective way to remove mismatches.

Matching between multimodal remote sensing images is a challenging task. In recent years, an increasing number of methods have been proposed. For example, Zhu et al. [46] introduced a robust model tackling the difficulty of identifying feature correspondences between multimodal images due to the significant differences both in radiation and geometry. By employing rotation-invariant feature descriptors, the method captures the rotational invariance of the key points, thereby facilitating stable feature matching. To address the problem of scale and rotation variations between multimodal remote sensing images, Ye et al. [47] proposed a novel descriptor and a fast normalised cross-correlation similarity measure. Their approach showed excellent performance in multimodal remote sensing image pairing.

Although great achievements have been made in image matching for remote sensing, how to simultaneously improve the matching accuracy and efficiency is still a noteworthy issue, especially for practical applications. In addition, no clear advancement has so far been seen in the object remote sensing image matching.

## 3. Proposed Method

**Figure 4:**
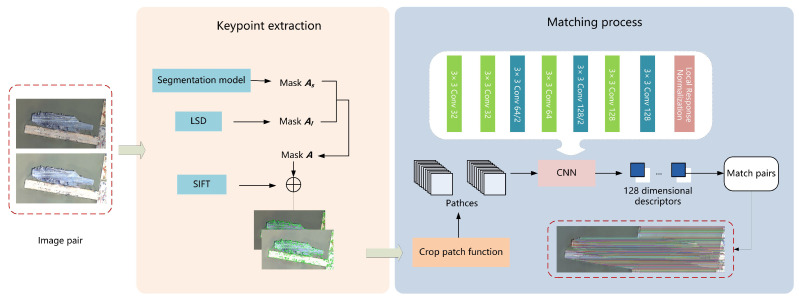
Diagram of the proposed image matching method. The keypoints used for matching are obtained by sifting the SIFT keypoints with mask A obtained by the intersection of marks $A_{s}$ and $A_{l}$, which are generated from segmentation and the line feature detector, respectively. The final selected keypoints are used with our cropping patch function to generate patches, which are then processed by the CNN descriptor for matching image pairs.

In this section, we firstly describe how to extract keypoints using line features in our solution and then present the developed image matching strategy, see Figure 4 for the diagram of the proposed method.

### 3.1. Keypoints Extraction with Line Features

The number of keypoints extracted by SIFT can be quite large, and lots of the keypoints extracted are unessential and/or leading to false matches. Meanwhile, using that high number of keypoints for image matching can be difficult for some close points and quite demanding in terms of computation cost. To apply the image matching algorithm on resource-limited satellites, we argue that matching images with a smaller number of points could be more feasible for applications. Inspired by this claim, below we present our designed algorithm which needs a much less number of keypoints by the help of line features.

There are many simple but reliable line detectors to extract line features, e.g., HoughP [48], fast line detector (FLD) [48] and line segment detector (LSD) [49]. We find that the line features detected by HoughP are almost horizontal lines, and the FLD detector is more likely to detect features in the background. In contrast, LSD can extract almost all line features while being less noisy. In our framework, we use LSD to extract line features, which will then be used to extract keypoints. Let $L_{\mathrm{LSD}}$ be the set of line features extracted by LSD, containing all the points on the detected lines. Let $S_{\mathrm{LSD}}\subset L_{\mathrm{LSD}}$ be the set of keypoints used for matching. In particular, $S_{\mathrm{LSD}}$ can be a set containing the two endpoints (according to the Krein–Milman theorem) of every detected line in $L_{\mathrm{LSD}}$.

**Figure 5:**
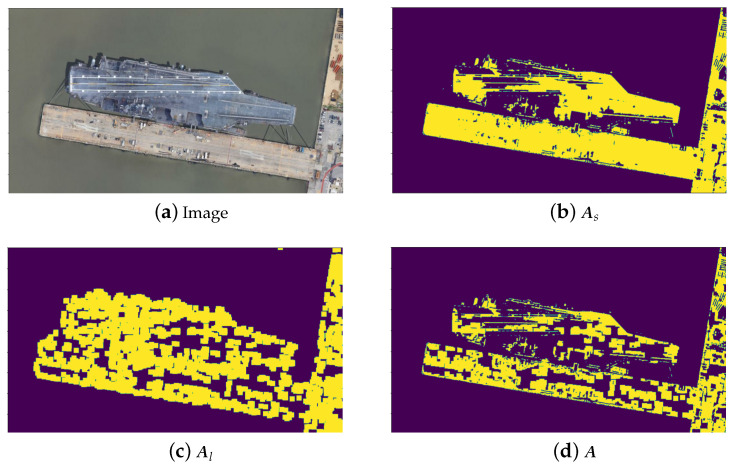
Example of different mask matrices. (**a**): the given image; (**b**): mask $A_{s}$ obtained by the segmentation technique; (**c**): mask $A_{l}$ obtained by utilising the line features; and (**d**): mask A, including the line features but with the background information removed.

We firstly generate a mask matrix $A_{l}$ with the same size as a channel of the given image I (i.e., a colour image with red, green and blue channels) using the line features in $S_{\mathrm{LSD}}$. In detail, $A_{l}$ is formed by setting its elements with positions in $S_{\mathrm{LSD}}$ to 1 otherwise 0, and then the elements with value 1 are expanded by applying the dilation operator so that $A_{l}$ can cover more areas and therefore be more inclusive. In the scene which contains ships for example, the ship information is more important than that in the background. Therefore, it is reasonable to tailor the mask $A_{l}$ by removing the background information. This can be achieved by generating another mask matrix say $A_{s}$, which is obtained by segmenting the most interesting areas from the images. The segmentation model can be trained on the remote sensing datasets. Then, the matrix mask, say A, considering both the line features and the important areas within the images can be obtained by

(1) $$A=A_{l}⊙A_{s},$$

where ⊙ represents pointwise multiplication. Note that it is straightforward to generate a set of keypoints say $S_{\mathrm{LSD}}^{A}$ using mask A, i.e., $S_{\mathrm{LSD}}^{A}$ is composed of all the points whose values are 1 in mask A. Figure 5 shows the difference of the mask matrices A, $A_{l}$ and $A_{s}$, from which we can see indeed mask A highlights the main areas (i.e., the ships) within the image.

Let $S_{\mathrm{SIFT}}$ be the set of keypoints obtained by SIFT. We suggest reducing the number of keypoints in $S_{\mathrm{SIFT}}$ by the set of keypoints obtained by line features $S_{\mathrm{LSD}}^{A}$. In other words, the set of keypoints we introduce for remote sensing image matching is defined as

(2) $$S=S_{\mathrm{SIFT}}\cap S_{\mathrm{LSD}}^{A}.$$

We name this keypoint extraction strategy LFKD (i.e., line features based keypoint detection). In our finding, mask $A_{s}$ and LSD can help to greatly remove keypoints that are not on the ship, see e.g., Figure 2 and Figure 5. This double-check can enhance the quality of the keypoints extracted, which will significantly benefit the subsequent matching task.

### 3.2. Matching Process

**Figure 6:**
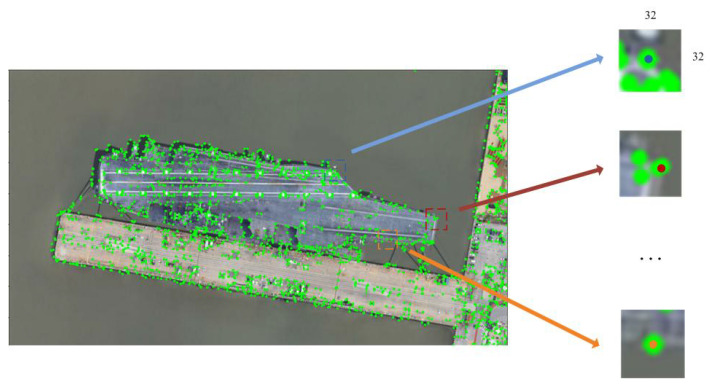
Image patch cropping corresponding to the keypoints in S.

We break the matching process into three stages. Firstly, for each given image $I_{i}$, we crop a patch centred on every keypoint in $S_{i}$ (here $S_{i}$ is the set of keypoints for image $I_{i}$) by using the warpAffine [48] function, which can achieve image transformation and cropping. The size of every patch is set to 32×32, see Figure 6.

Secondly, we use the trained CNN to get descriptors for every patch. To do so, the basic but effective CNN called HardNet, which includes a light backbone L2-Net and a strong loss function, is used. The descriptors generated in such a way can make the matching keypoints be selected easily in the matching step. Let ϕ(·) be the trained CNN and $I_{i}^{1},I_{i}^{2},\cdots ,I_{i}^{N_{i}}$ represent the $N_{i}$ cropped patches for image $I_{i}$. Then the descriptor say $d_{i}^{k}$ for patch $I_{i}^{k}$ can be obtained by $d_{i}^{k}=\phi \left(I_{i}^{k}\right)$. The descriptor set say $D_{i}$ for image $I_{i}$ can be formed by combining the descriptors of all the cropped patches, i.e.,

(3) $$D_{i}=\left\{d_{i}^{1},d_{i}^{2},\cdots ,d_{i}^{N_{i}}\right\}.$$

In the same way, the descriptor set can be created for any other images.

Finally, a set of matches say $M_{ij}$ between images $I_{i}$ and $I_{j}$ (i.e., bewteen their sets of keypoints $S_{i}$ and $S_{j}$) can be obtained by nearest neighbor between the descriptor set $D_{i}$ for image $I_{i}$ and the descriptor set $D_{j}$ for image $I_{j}$. For example, for a matching pair $\left(p,q\right)\in M_{ij}$, where $p\in S_{i}$ and $q\in S_{j}$, then *p* and *q* satisfy

(4) $$\begin{array}{cc} q & =\arg\min_{k}\{∥d_{i}^{p}-d_{j}^{1}∥,\cdots ,∥d_{i}^{p}-d_{j}^{k}∥,\cdots ,∥d_{i}^{p}-d_{j}^{N_{j}}∥\},\\ p & =\arg\min_{k}\{∥d_{i}^{1}-d_{j}^{q}∥,\cdots ,∥d_{i}^{k}-d_{j}^{q}∥,\cdots ,∥d_{i}^{N_{i}}-d_{j}^{q}∥\}. \end{array}$$

The matching set $M_{ij}$ can then be formed by finding all the matching pairs of the keypoints of images $I_{i}$ and $I_{j}$ satisfying Equation (4). For implementation, we find $M_{ij}$ by using the OpenCV built-in function BFMatcher.

In sum, the diagram of our developed image matching strategy is given in Figure 4. It mainly contains two parts, i.e., keypoints extraction and matching process. For a pair of images, the masks $A_{s}$ and $A_{l}$ for each of them are firstly generated by using an image segmentation method and LSD line detector, respectively. Mask A is obtained by taking the intersection of masks $A_{s}$ and $A_{l}$. The keypoints are the intersection of the ones selected by mask A and the ones selected by using SIFT. The keypoints are then used for the matching process. Each image is cropped into a bunch of patches centred around the keypoints, which will be used to form a descriptor set by using the trained CNN model. The descriptor sets for both images are finally used to obtain the matching pairs. The complete matching process is summarised in Algorithm 1.
**Algorithm 1:** Matching algorithm for remote sensing utilising line features**Input**: an image pair $\{I_{i}$, $I_{j}\}$**Output**: the matching set $M_{ij}$**1** Obtain mask A from Equation (1);**2** Get the sets of keypoints $S_{i}$ and $S_{j}$ using Equation  (2) for $I_{i}$ and $I_{j}$, respectively;**3** Get ϕ(·) from the trained CNN;**4** Crop image patches for $I_{i}$ and $I_{j}$;**5** Obtain the descriptors $D_{i}$ and $D_{j}$ by using Equation (3) for $I_{i}$ and $I_{j}$, respectively;**6** Find all the matching pairs by using Equation (4) and form the matching set $M_{ij}$.

## 4. Results

In this section, we first introduce the used datasets, i.e., one is from our own and the other two are publicly available, and the evaluation metrics. Thorough validation of the performance of the proposed method (shortened as LFKD for simplicity) and comparison with the relevant state-of-the-art methods are conducted afterwards, including detailed ablation study.

### 4.1. Data

#### 4.1.1. Dataset NWPU VHR-10

The NWPU VHR-10 dataset [50], released by Northwestern Polytechnical University in 2016, is a remote sensing dataset for space object detection. It contains ten different ground objects, i.e., aircraft, ships, oil tanks, ballpark, tennis courts, basketball courts, track and field fields, ports, bridges and cars, with a total of 3651 objects. There are 800 images in the dataset, where 715 RGB images were obtained from Google Earth with a spatial resolution range from 0.5 to 2 m, and 85 sharpened color infrared images were obtained from Vaihingen data with a spatial resolution of 0.08 m. The publisher annotated all images in the form of horizontal annotation boxes and provided annotation information making it an easy to train and test. We choose the images which include ship objects to test our proposed remote sensing ship matching method and make comparison.

#### 4.1.2. Dataset HRSC

The HRSC dataset, extracted from six important ports from Google Earth, was released by Northwestern Polytechnical University in 2016 [51]. It includes 1680 images with sizes from 300×300 to 1500×900 pixels. It owns unique characteristics, including a large number of ship images in different types, making it suitable for the tasks of this paper.

#### 4.1.3. Our Dataset

Our own dataset consists of twelve remote sensing images for three kinds of ships (i.e., aircraft carrier, cargo ship and submarine), which is created for testing remote sensing object matching methods. Compared with the other publicly available datasets, the images in our dataset contain the objects clearly. In each ship category, three factors are considered—illumination, size of the matching object and angle. Moreover, we also consider images containing multiple objects (see the submarine category). All the images are obtained from Google Earth. They are colourful and are of size 1600×900×3. During test, we choose one image as the benchmark in each class, and match it with others. Table 1 shows some samples from our own dataset, with brief description of the characteristics of each category.

### 4.2. Evaluation Metric

We use precision ρ to evaluate the methods’ performance, i.e.,

(5) $$\rho =N_{c}/N_{a},$$

where $N_{c}$ and $N_{a}$ refer to the number of the correct matches and the number of all matches, respectively. To evaluate the methods’ resource consumption, we use *M* and *T* to represent the memory cost and testing time, respectively, i.e.,

(6) $$M=M_{\mathrm{end}}-M_{\mathrm{start}},\;T=T_{\mathrm{end}}-T_{\mathrm{start}}\;,$$

where the subscripts ${}_{\mathrm{start}}$ and ${}_{\mathrm{end}}$ respectively represent the beginning and end of the methods’ memory occupation and running time during the matching process.

### 4.3. Results

We now show the results of our proposed method on the created remote sensing dataset and make comparison with SIFT and SIFT+CNN, where HardNet [52] is used for CNN in the experiment. More experiments on the publicly available datasets are given in the next subsection.

**Figure 7:**
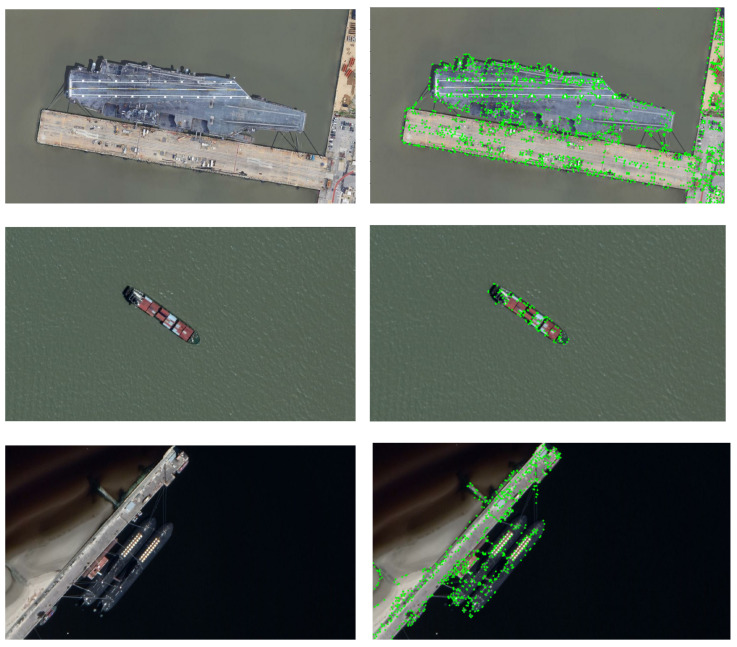
Keypoints obtained by the proposed method. The first and second columns are the original images and the keypoints obtained by our method imposed on the original images, respectively.

**Figure 8:**
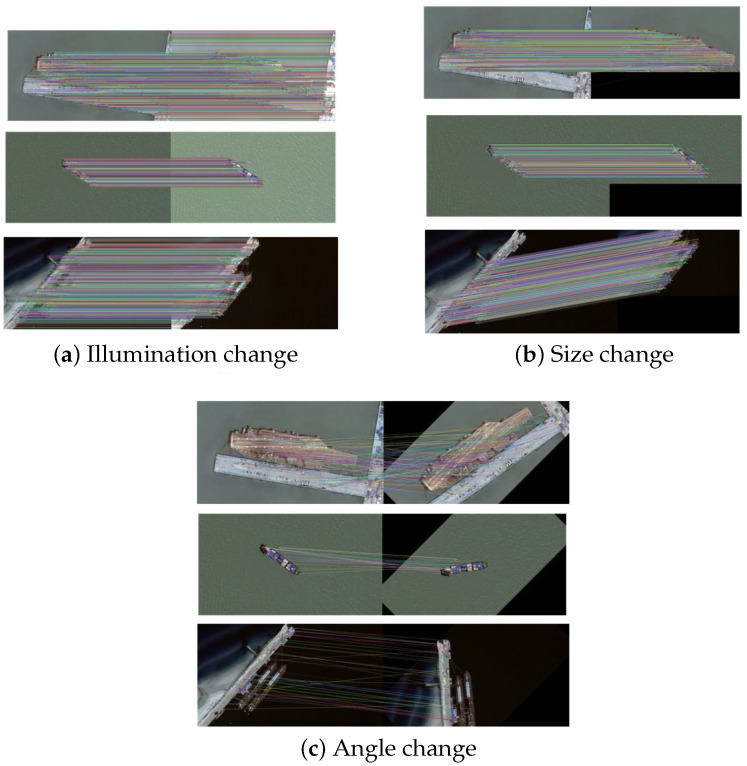
Ship matching results by using our method. (**a**–**c**) present the matching results on image pairs with changes in illumination, size and angle, respectively.

The quantitative results are given in Table 2, showing the number of correct matches $N_{c}$, the number of all matches $N_{a}$, and the precision ρ of each method. The results in Table 2 show that SIFT always extracts a large number of keypoints including a lot of wrong matches at the same time. Using CNN to get descriptors can significantly reduce the number of matches and incorrect matches and deliver a big improvement in precision. Our method can further improve the precision compared with SIFT and SIFT+CNN and achieve the best performance, see also the last row of Table 2 showing the average precision of each method. Moreover, Figure 7 shows the keypoints extracted by our method from some images, indicating that these extracted keypoints are indeed reasonable with good distribution without dense point clusters. This kind of keypoint distribution could effectively assist the method to avoid wrong matches caused by the positions of keypoints that are too close. Figure 8 showcases the matching results of our method on some image pairs with changes in illumination, size and angle, respectively, demonstrating its ability in removing most of the false matches on the background by using SIFT.

Through the result of these experiments, we found that our method also displays quite a low resource consumption and running time with high matching accuracy, and thus it could be equipped on satellites or other resource-limited scenes, see Table 3. In particular, Table 3 shows the time (ms) and memory (MB) spent of matching two images. It presents our keypoint extraction method can achieve ∼50% test speed boost and ∼30% memory saving with or without involving CNN.

### 4.4. Ablation Study

Ablation evaluations are conducted in this section to further demonstrate the effectiveness and robustness of our method. Firstly, we test all the methods on two different publicly available datasets, i.e., NWPU VHR-10 [50] and HRSC [51]. We choose three images about ship from each dataset and apply the same change to them in terms of illumination, size and angle. Table 4 shows the average precision of each method in different cases, indicating the superior performance of our method. We also match different ships and show the result in Table 5. We choose five different kinds of ships and all these images are from the HRSC dataset. Table 5 shows that the precision of matching two different ships is lower than matching the same one, which is reasonable, and our method performs the best.

Furthermore, we also test our method with different CNN backbones, i.e., SOSNet [53] and CSNet [54], and make a comparison with SIFT being equipped with these CNN models. The quantitative comparison in precision is shown in Table 6, which further validates that our method can indeed improve the matching precision in different settings and datasets in a robust manner.

Finally, to make the experiments more complete, we also compare our method with many other common but popular keypoint extraction methods in image matching on our dataset. Table 7 shows the performance comparison between methods SURF+CNN, ORB+CNN, Harris+CNN and Superpoint+CNN and ours in terms of effectiveness and efficiency. From Table 7, we can see that our method achieves nearly the same accuracy as the state-of-the-art result obtained by Superpoint+CNN (i.e., 0.972 vs. 0.973), but with significantly faster testing time and lower memory consumption, demonstrating our method’s suitability for remote sensing scenes, particularly in resources-limited satellites.

### 4.5. Discussion

We have shown that the proposed matching method can achieve significant improvement in running time and memory saving with great matching accuracy by combining line features and SIFT. Applying the proposed method and SIFT-related models to a resource-limited background (i.e., using limited computer parameters to simulate satellite environments), we found that our method is both more accurate and far more efficient, as demonstrated in different datasets.

Our method can achieve good performance by combining line features and CNN at multiple levels. This is comparable to previous results [52,53,54]. In comparison to previous studies, our method exhibited two-fold principal advantages in matching precision and efficiency. We found that our keypoint detector can extract features in important locations like edges and corners, and can avoid the dense distribution of keypoints compared with other detectors. That is beacause our method uses strong line features to filter out large number of unnecessary keypoints rather than only considering gradient. We therefore believe that more reasonable keypoints can help improve the matching performance, which is consistent with the conclusion of some existing research. In addition, we found that the precision achieved by methods compared on our dataset is higher than that from the publicly available datasets (see Table 2 and Table 4). This is because our dataset owns obvious objects without complicated background. That can help the methods mitigate the number of wrong matches. Most importantly, our method targets applications more, particularly in resource-limited satellites, for helping users build a powerful space-based information system. Some existing works [44,45] focus on matching two whole images using some special structures like attention model to improve the matching accuracy. Compared with the above ways, the method proposed in this paper firstly pays more attention to object matching with an end-to-end matching solution.

## 5. Conclusions and Future Work

In this paper, we created a new remote sensing dataset for ship matching and proposed a new method by exploiting line features for the matching task. The proposed method is simple yet effective for the remote sensing ship matching task, and is particularly designed for the use on resource-limited satellites. We showed that meaningful keypoints rather than their large number could boost the matching results dramatically. Thorough experiments including ablation study demonstrated that the proposed method can obtain better performance such as matching accuracy (i.e., 0.972 in our dataset, 0.954 and 0.889 in public datasets), 50% test speed boost and 30% memory saving. In the future, it is of great interest to work on different kinds of object matching, e.g., applying the proposed method to more remote sensing object matching applications. Moreover, it may be worthwhile to take an exploration in quickly determining the object category using the principle of the highest matching in the data scarcity scenario.

## Figures and Tables

**Table 1:** Samples from our own ship dataset.

Class	Image	Characteristic
Aircraft carrier	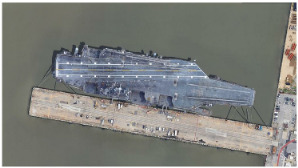	Large size, near harbors or in the ocean
Cargo ship	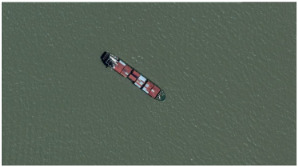	Small size, always in the ocean
Submarine	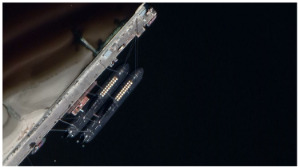	Small size, always near harbors

**Table 2:** Quantitative matching performance comparison on the created dataset.

Category		SIFT			SIFT+CNN			LFKD			LFKD+CNN		
		$N_{c}$	$N_{A}$	ρ	$N_{c}$	$N_{A}$	ρ	$N_{c}$	$N_{A}$	ρ	$N_{c}$	$N_{A}$	ρ
Aircraft Carrier	illumination	14,188	15788	0.898	3346	3446	0.970	7821	7353	0.940	3353	3441	**0.974**
	size	17,287	18,083	0.955	3412	3421	0.997	8705	8454	0.971	3457	3461	**0.998**
	angle	14,962	16,299	0.917	131	135	**0.970**	8484	8117	0.956	128	132	0.969
Cargo Ship	illumination	27,179	27434	0.990	4230	4235	0.998	630	590	0.936	539	539	**1**
	size	16,182	16,683	0.969	2255	2268	0.994	694	684	0.985	535	535	**1**
	angle	14,022	19,146	0.733	35	44	0.795	539	574	0.939	29	30	**0.996**
Submarine	illumination	12,904	13,987	0.922	3774	3837	0.983	2528	2397	0.948	1880	1915	**0.984**
	size	10,468	11,733	0.892	3607	3623	0.995	2664	2536	0.951	1950	1955	**0.997**
	angle	8465	14344	0.590	31	40	0.775	2017	1779	**0.882**	36	43	0.837
Average		0.874			0.941			0.945			**0.972**		

**Table 3:** Matching efficiency comparison in terms of running time and memory.

Method	Memory Cost (*M*)	Test Time (*T*)
SIFT	107 MB	69 ms
SIFT+CNN	532 MB	179 ms
LFKD	64 MB	42 ms
LFKD+CNN	313 MB	138 ms

**Table 4:** Matching precision comparison on datasets NWPU VHR-10 and HRSC.

Category		SIFT	SIFT+CNN	LFKD	LFKD+CNN
NWPU VHR-10	illumination	0.991	0.983	**0.992**	0.985
	size	0.873	0.885	0.879	**0.894**
	angle	0.720	0.837	0.868	**0.954**
HRSC	illumination	0.975	0.980	0.977	**0.982**
	size	0.891	**0.900**	**0.900**	0.895
	angle	0.815	0.850	0.841	**0.889**

**Table 5:** Matching precision comparison on different ships.

Category	SIFT	SIFT+CNN	LFKD	LFKD+CNN
1	0.879	0.915	0.908	**0.920**
2	0.729	0.836	0.827	**0.898**
3	0.902	0.947	0.939	**0.964**
4	0.835	**0.886**	0.836	0.844
5	0.895	0.926	0.931	**0.939**

**Table 6:** Comparison in precision between SIFT and our method with different CNN models (i.e., SOSNet and CSNet).

Method		SIFT+ SOSNet	LFKD+ SOSNet	SIFT+ CSNet	LFKD+ CSNet
Aircraft Carrier	illumination	0.971	**0.974**	0.971	0.973
	size	0.995	**0.997**	0.995	**0.997**
	angle	**0.970**	**0.970**	0.959	0.965
Cargo Ship	illumination	0.997	0.997	0.998	**1**
	size	0.997	**1**	0.994	**1**
	angle	0.801	0.993	0.792	**0.998**
Submarine	illumination	0.980	0.979	**0.988**	0.982
	size	0.995	**0.996**	**0.996**	0.995
	angle	0.760	0.825	0.779	**0.838**

**Table 7:** Comparison between our method with other keypoint extraction methods in image matching with CNN in terms of precision, memory cost and testing time.

Method	Precision (ρ)	Memory Cost (*T*)	Testing Time (*M*)
SURF+CNN	0.956	352 MB	162 ms
ORB+CNN	0.906	328 MB	159 ms
Harris+CNN	0.968	375 MB	154 ms
Superpoint+CNN	**0.973**	526 MB	173 ms
LFKD+CNN	0.972	**313 MB**	**138 ms**

## Data Availability

Code and data will be available after the review process.

## Abbreviations

The following abbreviations are used in this manuscript:

DCSS	Deformed Contour Segment Similarity
CNN	Convolutional Neural Network
SIFT	Scale Invariant Feature Transform
SURF	Speeded Up Robust Features
ORB	Oriented Fast and Rotated Brief
LoG	Laplacian of Gaussian
DoG	Difference of Gaussians
FLD	Fast Line Detector
LSD	Line Segment Detector

## References

[1] Li D., Shen X., Chen N., Xiao Z. (2017). Space-based information service in internet plus era. Sci. China Inf. Sci..

[2] Liu T., Yang Z., Marino A., Gao G., Yang J. (2020). Robust CFAR detector based on truncated statistics for polarimetric synthetic aperture radar. IEEE Trans. Geosci. Remote Sens..

[3] Shao Z., Wang L., Wang Z., Du W., Wu W. (2019). Saliency-aware convolution neural network for ship detection in surveillance video. IEEE Trans. Circuits Syst. Video Technol..

[4] Arandjelovic R., Gronat P., Torii A., Pajdla T., Sivic J. NetVLAD: CNN Architecture for Weakly Supervised Place Recognition. Proceedings of the CVPR 2016.

[5] Capobianco S., Forti N., Millefiori L.M., Braca P., Willett P. (2022). Recurrent encoder-decoder networks for vessel trajectory prediction with uncertainty estimation. IEEE Trans. Aerosp. Electron. Syst..

[6] Lowe D.G. (2004). Distinctive image features from scale-invariant keypoints. IJCV.

[7] Sarlin P.E., DeTone D., Malisiewicz T., Rabinovich A. Superglue: Learning Feature Matching with Graph Neural Networks. Proceedings of the IEEE/CVF Conference on Computer Vision and Pattern Recognition.

[8] Bay H., Tuytelaars T., Gool L.V. (2006). Surf: Speeded up Robust Features. Computer Vision–ECCV 2006, Proceedings of the 9th European Conference on Computer Vision, Graz, Austria, 7–13 May 2006.

[9] Ye Y., Bruzzone L., Shan J., Bovolo F., Zhu Q. (2019). Fast and robust matching for multimodal remote sensing image registration. IEEE Trans. Geosci. Remote Sens..

[10] Li J., Hu Q., Ai M. (2019). RIFT: Multi-modal image matching based on radiation-variation insensitive feature transform. IEEE Trans. Image Process..

[11] Hartley R.I. (1997). In defense of the eight-point algorithm. IEEE Trans. Pattern Anal. Mach. Intell..

[12] Moravec H.P. Towards Automatic Visual Obstacle Avoidance. Proceedings of the IJCAI1977.

[13] Harris C.G., Stephens M.J. A Combined Corner and Edge Detector. Proceedings of the Alvey Vision Conference.

[14] Rosten E. (2006). Machine Learning for High-Speed Corner Detection. Computer Vision—ECCV 2006, Proceedings of the 9th European Conference on Computer Vision, Graz, Austria, 7–13 May 2006.

[15] Rublee E., Rabaud V., Konolige K., Bradski G.R. ORB: An Efficient Alternative to SIFT or SURF. Proceedings of the 2011 International Conference on Computer Vision.

[16] Smith S.M., Brady J.M. (1997). SUSAN—A New Approach to Low Level Image Processing. Int. J. Comput. Vis..

[17] Canny J.F. (1987). A computational approach to edge detection. Readings Comput. Vis..

[18] Perona P., Malik J. (1990). Scale-space and edge detection using anisotropic diffusion. IEEE Trans. Pattern Anal. Mach. Intell..

[19] Sandu I.R., Antoniu E. (1993). Laplacian-of-Gaussian Filter in Edge Detection. Tensor.

[20] Lindeberg T. (1996). Scale-Space Theory: A Framework for Handling Image Structures at Multiple Scales. https://cds.cern.ch/record/400314/files/p27.pdf.

[21] Derpanis K.G. (2004). The Harris Corner Detector. Symposium Svenska Sllskapet Fr Bildanalys.

[22] Shi J., Tomasi C. (2002). Good Features to Track. Proceedings of the IEEE Conference on Computer Vision and Pattern Recognition.

[23] John C. (1986). A computational approach for edge detection. IEEE Trans. Pattern Anal. Mach. Intell..

[24] Quinlan J.R. (1986). Induction of decision trees. Mach. Learn..

[25] Lepetit V., Fua P. (2006). Keypoint Recognition Using Randomized Trees. IEEE Trans. Pattern Anal. Mach. Intell..

[26] Strecha C., Lindner A., Ali K., Fua P., Süsse H. (2009). Training for Task Specific Keypoint Detection.

[27] Hartmann W., Havlena M., Schindler K. Predicting Matchability. Proceedings of the IEEE Conference on Computer Vision and Pattern Recognition (CVPR).

[28] Verdie Y., Yi K., Fua P., Lepetit V. TILDE: A Temporally Invariant Learned DEtector. Proceedings of the IEEE Conference on Computer Vision and Pattern Recognition (CVPR).

[29] Zitová B., Flusser J. (2003). Image Registration Methods: A Survey. Image Vis. Comput..

[30] Li Z., Mahapatra D., Tielbeek J.A.W., Stoker J., Vliet L.J.V., Vos F.M. Image Registration Based on Autocorrelation of Local Structure. Proceedings of the Interspeech.

[31] Reddy B.S., Chatterji B.N. (1996). An FFT-based technique for translation, rotation, and scale-invariant image registration. IEEE Trans. Image Process..

[32] Lin W.Y., Liu S., Jiang N., Do M.N., Lu J. RepMatch: Robust Feature Matching and Pose for Reconstructing Modern Cities. Proceedings of the European Conference on Computer Vision.

[33] Lazaridis G., Petrou M. (2006). Image registration using the Walsh transform. IEEE Trans. Image Process..

[34] Wei S.D., Pan W.H., Lai S.H. Efficient NCC-Based Image Matching Based on Novel Hierarchical Bounds. Proceedings of the Pacific Rim Conference on Multimedia: Advances in Multimedia Information Processing.

[35] Wu Q., Xu G., Cheng Y., Wang Z., Li Z. (2021). Deformed contour segment matching for multi-source images. Pattern Recognit..

[36] Wang A., Pruksachatkun Y., Nangia N., Singh A., Michael J., Hill F., Levy O., Bowman S.R. (2019). SuperGLUE: A Stickier Benchmark for General-Purpose Language Understanding Systems. arXiv.

[37] Wang G., Luo C., Xiong Z., Zeng W. Spm-tracker: Series-parallel matching for real-time visual object tracking. Proceedings of the IEEE/CVF Conference on Computer Vision and Pattern Recognition.

[38] Liao Y., Liu S., Wang F., Chen Y., Qian C., Feng J. Ppdm: Parallel point detection and matching for real-time human-object interaction detection. Proceedings of the IEEE/CVF Conference on Computer Vision and Pattern Recognition.

[39] Cheng L., Li M., Liu Y., Cai W., Chen Y., Yang K. (2012). Remote sensing image matching by integrating affine invariant feature extraction and RANSAC. Comput. Electr. Eng..

[40] Zhu H., Jiao L., Ma W., Liu F., Zhao W. (2019). A Novel Neural Network for Remote Sensing Image Matching. IEEE Trans. Neural Netw. Learn. Syst..

[41] Sedaghat A., Mohammadi N. (2019). Illumination-Robust remote sensing image matching based on oriented self-similarity. ISPRS J. Photogramm. Remote Sens..

[42] Su J., Lin X., Liu D. (2007). Change detection algorithm for remote sensing images based on object matching. Qinghua Daxue Xuebao/J. Tsinghua Univ..

[43] Hou B., Ren Z., Zhao W., Wu Q., Jiao L. (2019). Object Detection in High-Resolution Panchromatic Images Using Deep Models and Spatial Template Matching. IEEE Trans. Geosci. Remote. Sens..

[44] Li L., Liu M., Ma L., Han L. (2022). Cross-Modal feature description for remote sensing image matching. Int. J. Appl. Earth Obs. Geoinf..

[45] Fan X., Xing L., Chen J., Chen S., Bai H., Xing L., Zhou C., Yang Y. (2022). VLSG-SANet: A feature matching algorithm for remote sensing image registration. Knowl. Based Syst..

[46] Zhu B., Yang C., Dai J., Fan J., Qin Y., Ye Y. (2023). R_2_FD_2_: Fast and Robust Matching of Multimodal Remote Sensing Images via Repeatable Feature Detector and Rotation-Invariant Feature Descriptor. IEEE Trans. Geosci. Remote Sens..

[47] Ye Y., Zhu B., Tang T., Yang C., Xu Q., Zhang G. (2022). A Robust Multimodal Remote Sensing Image Registration Method and System Using Steerable Filters with First- and Second-order Gradients. ISPRS J. Photogramm. Remote Sens..

[48] Bradski G., Kaehler A., OpenCV Dr. Dobb’s Journal of Software Tools.

[49] Von Gioi R.G., Jakubowicz J., Morel J.M., Randall G. (2008). LSD: A fast line segment detector with a false detection control. IEEE Trans. Pattern Anal. Mach. Intell..

[50] Cheng G., Han J., Zhou P., Guo L. (2014). Multi-class geospatial object detection and geographic image classification based on collection of part detectors. ISPRS J. Photogramm. Remote Sens..

[51] Liu Z., Yuan L., Weng L., Yang Y. A High Resolution Optical Satellite Image Dataset for Ship Recognition and Some New Baselines. Proceedings of the International Conference on Pattern Recognition Applications and Methods.

[52] Mishchuk A., Mishkin D., Radenovic F., Matas J. (2017). Working hard to know your neighbor’s margins: Local descriptor learning loss. Adv. Neural Inf. Process. Syst..

[53] Tian Y., Yu X., Fan B., Wu F., Heijnen H., Balntas V. Sosnet: Second order similarity regularization for local descriptor learning. Proceedings of the CVPR 2019.

[54] Zagoruyko S., Komodakis N. Learning to compare image patches via convolutional neural networks. Proceedings of the IEEE Conference on Computer Vision and Pattern Recognition.

